# The association between maternal lifetime interpersonal trauma experience and perceived mother-infant bonding

**DOI:** 10.1016/j.jad.2021.06.069

**Published:** 2021-11-01

**Authors:** Tessel Annejo Kolk, Selina Nath, Louise Michele Howard, Susan Pawlby, Georgia Lockwood-Estrin, Kylee Trevillion

**Affiliations:** a Section of Women's Mental Health, Institute of Psychiatry, Psychology and Neuroscience, King's College London, De Crespigny Park, London SE5 8AF, UK.; b Division of Psychological Medicine, Institute of Psychiatry, Psychology & Neuroscience, King's College London, De Crespigny Park, London SE5 8AF, UK; c Henry Wellcome Building, Centre for Brain and Cognitive Development, Birkbeck College, 32 Torrington Square, London WC1E 7JL, UK

**Keywords:** Mother-infant bonding, Lifetime trauma, Depression, Perinatal

## Abstract

**Background:**

Interpersonal traumas are common among expectant and new mothers and are found to have considerable impacts on women's mental health. These experiences may disrupt maternal perceptions of the mother-infant relationship, which is essential for healthy infant development, but findings are inconsistent. This study aims to explore associations between lifetime interpersonal traumas and their impact on self-reported mother-infant bonding.

**Methods:**

Secondary data analysis of a representative cohort of 453 women attending at a South London maternity service. Lifetime interpersonal trauma experience and its association with self-reported mother-infant bonding (Postpartum Bonding Questionnaire) was assessed in uni- and multivariable linear regressions, the latter adjusted to account for antenatal depressive and posttraumatic symptoms, measured using the Edinburgh Postnatal Depression Scale and Posttraumatic Stress Disorder Scale, and key sociodemographic risk factors.

**Results:**

Maternal lifetime trauma was not associated with perceived difficulties in mother-infant bonding at three months postnatal; however antenatal depressive symptoms, both with continuous EPDS score (0.33, 95% CI 0.17–0.50, p<0.001) and clinical cut-off ≥13 (4.26, 95% CI 2.02–6.49, p<0.001) were associated with self-reported bonding difficulties.

**Limitations:**

The composite trauma measurement did not allow for a comprehensive assessment of individual trauma types.

**Conclusions:**

There was no evidence for a link between maternal lifetime trauma experiences and self-reported bonding difficulties. However, an association between antenatal depressive symptoms and perceived postpartum bonding impairment was found. This highlights the importance of identification and treatment of depressive symptoms during pregnancy and offering women support in facilitating a positive mother-infant relationship.

## Highlights

• Prevalence of lifetime trauma experience in a community birth cohort is 32%.
• Mothers with experience of lifetime trauma have higher depressive symptoms.
• Antenatal depressive symptoms are a risk factor for self-reported impaired bonding.

## 1 Introduction

### 1.1 Interpersonal trauma, the perinatal period and maternal-infant bonding

Interpersonal trauma can be defined as “an exposure to actual or threatened death, serious injury or sexual violence”; such traumas can be either directly experienced or witnessed (American Psychiatric Association, 2013). Women are much more likely than men to experience multiple and repeated forms of interpersonal trauma (Howard et al., 2010; Walby and Allen, 2004), including intimate partner violence (IPV) and sexual violence (Holt et al., 2008; Kessler et al., 2017). Indeed, global surveys indicate that up to 71% of women have experienced physical or sexual partner violence in their lifetime (Garcia-Moreno et al., 2006) and evidence indicates that women of child-bearing age are highly likely to have experienced a traumatic event (Benjet et al., 2016; Gelaye et al., 2017).

Maternal-infant bonding is a construct that describes a mother's emotional connectedness to her infant (Nolvi et al., 2016). Impairments in maternal-infant bonding include delays in a mother's emotional responses towards her infant, anger and rejection (Brockington et al., 2001; Brockington et al., 2006). Parental sensitivity represents a form of maternal emotional connectedness and describes the responsiveness of mothers to the cues of their infants (Field, 2010; van Ee et al., 2016). Parental sensitivity is shown to be essential in the formation of positive mother-infant relationships and infant attachments (Field, 2010; Murray et al., 1996; Murray et al., 2010; Nolvi et al., 2016; Pereira et al., 2017). Trauma experience has been found to affect a mother's parenting (Burtchen et al., 2016; Delker et al., 2014; Iyengar et al., 2014; Madigan et al., 2019; Savage et al., 2019), sensitivity (van Ee et al., 2016) and perceived bonding (Kita et al., 2016; Lara-Cinisomo et al., 2018; Muzik et al., 2013; Seng et al., 2013). For example, mothers affected by trauma in childhood have shown harsher parenting styles, increased hostility and intrusiveness towards their infant (Savage et al., 2019). Such responses may be due to mental health disturbances arising as a consequence of interpersonal trauma, which negatively affect mothers’ parenting behaviours (Erickson et al., 2019; Madigan et al., 2019; Murray et al., 2010). Trauma may also affect parental sensitivity via associated altered perceptions that trauma-exposed mothers have of their infant and altered development of maternal emotional regulation (Iyengar et al., 2014). Moreover, trauma experience may impede the ability of a parent to provide steady and positive care, which is essential for the self-regulatory development of their infants (Delker et al., 2014). In the longer term, perceived difficulties of the mother-infant relationship are known to affect the socio-emotional and cognitive development of the infant; self-reported poor bonding between mothers and their infants is associated with diminished adjustment to school at six years (Sturge-Apple et al., 2006) and internalising (fearful behaviour and social withdrawal) (Burtchen et al., 2016; Madigan et al., 2015) and externalising behaviours (physical aggression and disobeying rules) in children (Plant et al., 2017). Later, in early adolescence, these early experiences are found to be associated with the subsequent increased risk of development of depressive symptoms (Plant et al., 2017).

The literature on the influence of interpersonal trauma on self-reported maternal bonding difficulties largely focus on experiences of trauma in childhood; assessing the collective impact of neglect and emotional, physical and sexual abuse (Lehnig et al., 2019; Muzik et al., 2013; Seng et al., 2013). The impact of interpersonal trauma in early adulthood (Lockwood Estrin et al., 2019) and trauma in later life has been largely overlooked and warrants further exploration. Moreover, childhood trauma experiences are greatly associated with an increased risk of adulthood trauma experiences (Barnes et al., 2009; Choi and Sikkema, 2015; Dias et al., 2017; Frederick and Goddard, 2008), making it harder to observe the relative influence of distinct types of trauma in childhood versus adulthood.

### 1.2 Interpersonal trauma, mental health problems and the perinatal period

Global surveys indicate that forms of interpersonal trauma carry substantially higher risks for post-traumatic stress disorder in comparison to non-interpersonal forms of trauma (Kessler et al., 2017). In addition, interpersonal traumas represent a major risk factor for the development of depressive and anxiety symptoms (Chandan et al., 2019; Kessler et al., 2017; Khalifeh et al., 2015; Trevillion et al., 2012). Women are especially vulnerable to developing mental health difficulties following experiences of interpersonal trauma (Kessler et al., 2017). There is a large body of evidence examining the links between women's experience of childhood sexual abuse and the subsequent development of mental disorders (Spataro et al., 2004; Hailes et al., 2019), extending into the perinatal period (Akinbode et al., 2020). Other forms of abuse in childhood (i.e. physical and emotional abuse and neglect) and experiences of adulthood IPV are also associated with mental disorders in the perinatal period (Howard et al., 2013; Akinbode et al., 2020; Choi and Sikkema, 2015; Hailes et al., 2019; Kita et al., 2016; Mezey et al., 2005; Rich-Edwards et al., 2011; Spataro et al., 2004). There is increasing evidence that interpersonal trauma exposures across the lifetime increase an individual's risk of developing mental disorders (Shevlin et al 2013), and studies are inconclusive regarding which aforementioned trauma type are more predictive of mental distress (Choi and Sikkema, 2015).

The perinatal period is a challenging period for mothers and mental disorders in the perinatal period are one of the most common complications associated with pregnancy and motherhood (Howard et al., 2013; Howard et al., 2014; Martini et al., 2015; Twenge et al., 2003). Moreover, trauma experience and subsequent mental disorders represent a major public health issue in the perinatal period (Howard et al., 2014), affecting not only the functioning of mothers but also their parenting behaviours, parent-child bonding and the behavioural and emotional development of children (Burtchen et al., 2016; Erickson et al., 2019; Field, 2010; Lara-Cinisomo et al., 2018; Madigan et al., 2015; Martini et al 2015; Murray et al., 2010; Nath et al., 2019; Seng et al., 2011; Stein et al 2014).

### 1.3 Interpersonal trauma, mental health problems and mother-infant bonding

Research that examines the impact of adult experiences of trauma on early mother-child relationships is minimal and have usually focused on the impact of traumatic childbirth experiences (McDonald et al., 2011; Parfitt, 2009). Therefore, much of the potential impact of adulthood interpersonal traumatic experiences remains unexplored. Moreover, many existing study analyses examining the relationship between interpersonal trauma and perceived mother-infant bonding do not consistently adjust for crucial risk factors, such as mental health problems. There is a need, therefore, to examine the impact of lifetime (both in child- and/or adulthood) interpersonal trauma experiences on early mother-infant bonding patterns. For this reason, the primary aim of the current study was to assess the possible association between lifetime interpersonal trauma experiences and maternal self-reported bonding difficulties at three months postpartum. The analyses will control for important risk factors identified from the existing literature, i.e. antenatal depressive and posttraumatic stress symptoms, ethnicity, young maternal age, infant prematurity and education level (Hoffenkamp et al., 2012; Ionio et al., 2016; Lockwood Estrin et al., 2019; Loh and Vostanis, 2004; Sockol et al., 2014; Dubber et al., 2015), to examine their influence on perceived mother-infant bonding.

## 2 Methods

### 2.1 Study design and population

**Fig. 1:**
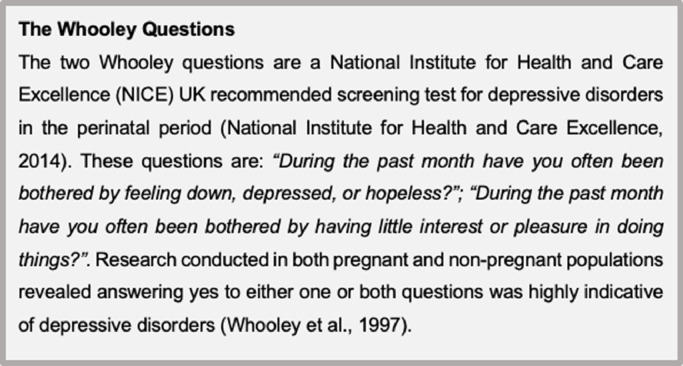
Description of the Whooley Questions.

This is a report of a secondary analysis of the WEll-being in pregNancy stuDY (WENDY) (Howard et al., 2018). WENDY is a cohort study of 545 pregnant women recruited between November 2014 and June 2016 at a large inner-city maternity service in South London, England. The WENDY study sought to examine the diagnostic accuracy of two depression-screening questions (the Whooley Questions, see Fig. 1), asked of all pregnant women residing in the UK at their first scheduled antenatal appointment with their midwifery practitioner (Howard et al., 2018; National Institute for Health and Care Excellence, 2014). This appointment is offered around 10 weeks’ gestation and is standard practice of care provided by the UK National Health Service. The two Whooley questions were compared to the Edinburgh Postnatal Depression Scale (EPDS), against the gold standard diagnostic Structured Clinical Interview DSM-IV. All women who answered ‘yes’ to either of the two depression-screening questions were considered positive for possible depressive disorders (Whooley et al., 1997).

**Fig. 2:**
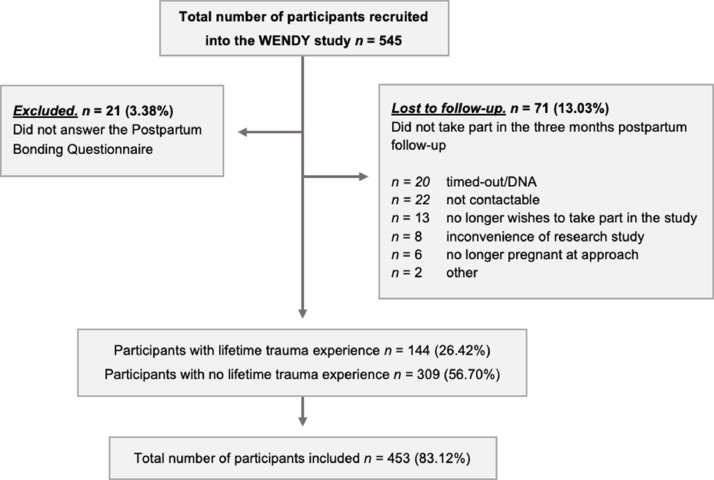
**Flowchart of participants through the study.** Note: DNA, did not attend.

All women who screened positive and a random sample of women who screened negative were approached for participation in the WENDY study. Those who were under the age of 16, those who had a previous maternity booking elsewhere, those who had a termination or miscarriage between the antenatal booking and the baseline research interview, or who had refused to answer the two depression-screening questions were excluded. A detailed baseline research interview was conducted with participants within a maximum of three weeks after the booking appointment. Two follow-up interviews were then scheduled with the participants at approximately 28 weeks’ gestation and three months postpartum. A total of 545 women were recruited into the WENDY study, of whom 287 screened positive and 258 negative on the depression-screening questions (see Howard et al. (2018) for full details on the study methodology). For this study, we excluded those who did not take part in the three months postpartum follow-up interview and those who did not answer the Postpartum Bonding Questionnaire (PBQ) at three months, see Fig. 2 for our final sample for analysis.

### 2.2 Measurements

For the purposes of this secondary analysis, the following measures from the WENDY study were analysed:

#### 2.2.1 Lifetime trauma composite measurement

A measure of composite lifetime trauma was generated by researchers at King's College London (Lockwood Estrin et al., 2019) using information on trauma gathered from the Composite Abuse Scale-short version (CAS(S) pregnancy version, the Posttraumatic Stress Disorder Scale (PDS®) and the Structured Clinical Interview DSM-IV Axis (SCID) PTSD module (First et al., 2002) at the baseline research interview.

**The CAS(S)** modified pregnancy version, is an 11-item self-administered questionnaire regarding physical, sexual and/or severe abusive behaviour of the partner in the year before and during pregnancy (Hegarty, 2007; Hegarty et al., 2005; Hegarty et al., 1999).

**The PDS®** is a questionnaire reporting lifetime exposure to traumatic events. It covers 49 items, is self-administered and based on the DSM-IV diagnosis of PTSD; the measure assesses the severity of posttraumatic stress symptoms in the past month (Foa et al., 1997). The PDS® records information about traumas including, but not limited to, the number of posttraumatic symptoms experienced, characteristics of the perpetrator (such as family or stranger) and the type of trauma experienced. The PDS® has previously been used in antenatal populations (Mezey et al., 2005). Questions one to 13 record information regarding trauma experienced, which were answered by all participants and included within the composite abuse measurement. In addition, this study used the PDS® questions 22 to 38 to quantify posttraumatic stress symptom severity in the past month, which were only answered by those recording at least one trauma on questions one to 13. Question scores ranged from zero to three from not feeling bothered by the trauma experience to thinking of it almost always. This study used the total score out of 48.

**The SCID** is a semi-structured diagnostic interview long-established in psychiatric research (First et al., 2002). **The SCID PTSD module** covers experiences of trauma throughout the lifetime of a person. All traumatic events volunteered by the person are documented together with the date of trauma and the age of the person at the time of trauma (used to categorise childhood abuse <16 years), as well as severity indicators for current PTSD.

Within the **lifetime trauma composite measurement,** trauma experiences could be categorised into:

• Physical abuse by known person or stranger (the latter only when categorised as traumatic);
• Sexual abuse (both attempted and completed) by known or unknown perpetrator;
• Sexual, physical and non-physical abuse by an intimate partner;
• Sexual, physical and non-physical abuse experienced in childhood (below the age of 16).

The lifetime trauma experiences of the participants could fall within one or multiple of these categories. These categories corresponded with the definitions used in the routine nationally representative crime survey of the general population in England and Wales, the Crime Survey for England and Wales (Office for National Statistics, 2018).

#### 2.2.2 Postpartum bonding questionnaire (PBQ)

The PBQ is a self-reported questionnaire and measures the perception of parental emotional bonding with their infant (Brockington et al., 2001). The PBQ was administered at the three months’ postpartum visit to assess for early mother-infant relationship difficulties. The PBQ includes 25 self-reported ratings on statements regarding maternal feelings and views towards her infant. The statements were rated from zero= “always” to five= “never” and the scoring reversed when the statement covers a negative feeling. The total PBQ scores range between zero to 125, with a higher score indicating increasingly perceived mother-infant bonding disruption (Brockington et al., 2001; Brockington et al., 2006). The total PBQ score was used as well as the four subscales; “impaired bonding”, “rejection and anger”, “anxiety about care” and “risk of abuse”. The PBQ (both total and subscales) have previously been used in postpartum populations (Edhborg et al., 2005; Reck et al., 2006; van Bussel et al., 2010) and in the context of maternal traumatic experiences (Muzik et al., 2013; Seng et al., 2013; Wittkowski et al., 2007).

#### 2.2.3 The Edinburgh Postnatal Depression Scale (EPDS)

Antenatal depressive symptoms were measured using the EPDS (Cox et al., 1987); a 10-item screening test for perinatal depression. For this study, we used the EPDS data collected at the baseline interview. The EPDS was included on a continuous scale and with a cut-off of 13 out of 30 to indicate highly symptomatic women, with a previously calculated weighted sensitivity of 59% and specificity of 94% (Gibson et al., 2009; Howard et al., 2018).

#### 2.2.4 Other measures

At the baseline interview socio-demographic characteristics were recorded. For the purposes of this paper, the following socio-demographic characteristics were examined: (1) maternal age, (2) ethnicity (“*white”, “black African/Caribbean/British”, “Asian/Asian British”, “Mixed/multiple ethnic groups”* and *“Arab/other ethnic groups”*), (3) highest level of education (“*none/school qualifications”, “college/diploma/higher certificate/training”* and *“university degree/postgraduate”*), (4) employment (*“employed/homemaker/student” and “unemployed/unable to work”,* (5) relationship status (*“single (e.g., in no relationship or widowed)” and “in a relationship (e.g., married or with a partner)”*), (6) current living status (*“alone”, with “spouse/partner”, “parent(s)”, “friends”, “other family members”, “acquaintance” and “other”*) and (7) household income. Obstetric data was also collected in the WENDY study, and the following variables were analysed in this study: (1) any previous children, (2) any previous miscarriages, (3) if the current pregnancy was planned or unplanned; (4) infant gender and (5) gestational age (GA) at birth in weeks.

### 2.3 Statistical analysis

Statistical analysis was carried out using Stata version 15.1 (StataCorp, 2017). First, descriptive statistics were calculated to examine the characteristics of those exposed to lifetime trauma compared to their non-exposed controls, using Pearson's chi-squared tests to discern significant differences for categorical data and Wilcoxon-Mann-Whitney test and t-test for continuous variables. Univariable regression analyses were run on the following variables chosen a priori from literature: EPDS, PDS® symptoms severity, sociodemographic (maternal age, ethnicity, education, employment, relationship status) and obstetric data (previous children, infant gender, infant GA at birth, pregnancy intention) and lifetime trauma experience, testing for any association with total and subscale PBQ scores through unadjusted linear regression. The relationship between lifetime trauma experience with total and subscale PBQ scores was then assessed through a multivariable linear regression, adjusted to all previously mentioned exposures. Sensitivity analysis was undertaken to investigate whether using a clinical cut-off point of ≥13 for the EPDS influenced the association between EPDS score (depressive symptoms) and the total PBQ outcome. Additionally, to account for possible skewedness of the PBQ score outcome, another sensitivity analysis was undertaken with transformed PBQ score data. Multivariable linear regression was then repeated using the transformed PBQ variable.

Sampling weights were created to account for bias induced by the stratified sampling using the *svy* command in Stata (Pickles et al., 1995). These weights were calculated based on the total number of women who screened positive and negative on the two case-finding depressive questions over the entire population of women who completed the first antenatal appointment during the same period that the WENDY study was running. Sampling weights were used in the adjusted multivariate linear regression analysis to account for the oversampling of Whooley positive participants; for details regarding the calculations for the sampling weights see Howard et al., 2018.

### 2.4 Missing data

PBQ were completed by 453 (95.57%) and missing for 21 (4.43%) participants. This was out of total 474 participants followed-up at three months postpartum (see Fig. 2). As the main outcome is the PBQ score, population characteristics were only described for those with the PBQ completed. Inverse probability weights were used to deal with potential bias introduced by missing PBQ data. It was determined that lower education was associated with missing data on the PBQ. The inverse probability weights were combined with the sampling weights for the adjusted multivariate analyses.

A total of 441 (97.35%) PBQ respondents had answered the EPDS and 12 (2.65%) had not. Regarding the PDS**®**, 429 (94.70%) participants had their answers noted, 24 (5.30%) participants had PDS® data missing. A total of three people had living status and employment data missing and infant gender and GA at birth of one participant was not recorded. None of the participants with missing data were included in the uni- and multivariable analyses in order to ensure complete case analysis was conducted.

## 3 Results

### 3.1 The WENDY study sample

Of the total 545 recruited women in the WENDY study, 474 (86.97%) took part in the three months’ postpartum follow-up and 71 (13.03%) women were lost to follow-up. Additionally, 21 (3.38%) out of all WENDY participants had the PBQ scores missing at the three months postpartum follow-up. A total of 453 (83.12%) participants were included in the study (see Fig. 2).

### 3.2 Sample characteristics

Of the 453 included participants, 144 (31.79%) reported experiencing trauma in their lifetime and 309 (68.21%) did not (see Table 1). Of those reporting a trauma, our composite trauma measurement recorded that 96 (66.67%) reported more than one incident of trauma experience (see details in Table 2).

**Table 1:** Study sample characteristics of those with experience of trauma in their lifetime and the unexposed control.

**Sample characteristics**				
**Characteristic**	**No experience of trauma**	**Lifetime trauma experience**	**Pearson's chi-squared test**	**Total**
	*n*=309 (68.21%)	*n*=144 (31.79%)	χ^2^(df, *n*)	*n*=453
	*n* (%)	*n* (%)	p-value	*n* (%)
				
Maternal Age (years old)*				
<20	0 (0.00)	4 (2.78)	χ^2^=13.90	4 (0.88)
20-29	72 (23.30)	47 (32.64)	(3, *n*=453)	119 (26.27)
30-39	214 (69.26)	83 (57.64)	p=0.003	297 (65.56)
≥40	23 (7.44)	10 (6.94)		33 (7.28)
				
**Ethnicity**				
White	179 (57.93)	72 (50.00)		251 (55.41)
Black African/Caribbean or black British	88 (24.48)	49 (34.03)	χ^2^=7.40	137 (30.24)
Asian/Asian British	13 (4.21)	6 (4.17)	(4, *n*=453)	19 (4.19)
Mixed/Multiple ethnic groups	8 (2.59)	10 (6.94)	p=0.116	18 (3.97)
Arab/Other ethnicity	21 (6.80)	7 (4.86)		28 (6.18)
				
Education (highest level achieved)*				
None/School qualifications	26 (8.41)	20 (13.89)	χ^2^=8.27	46 (10.15)
College/Diploma/Higher certificate/Training	103 (33.33)	60 (41.67)	(2, *n*=453)	163 (35.98)
University degree/Postgraduate	180 (58.25)	64 (44.44)	p=0.016	244 (53.86)
				
Employment*				
Employed/Homemaker/Student	268 (87.01)	104 (72.73)	χ^2^=13.79	372 (82.48)
			(1, *n*=451)	
Unemployed/Unable to work	40 (12.99)	39 (27.27)	p<0.001	79 (17.52)
				
Household income*				
<£15,000	26 (10.70)	31 (27.19)		57 (15.97)
£15,000 - £30,999	36 (14.81)	22 (19.30)	χ^2^=22.76	58 (16.25)
£31,000 - £45,999	45 (18.52)	9 (7.89)	(4, *n*=357)	54 (15.13)
£46,000 – £60,999	38 (15.64)	19 (16.68)	p<0.001	57 (15.97)
£61,000 or more	98 (40.33)	33 (28.95)		131 (36.69)
				
Relationship status*				
In a relationship^a^	284 (91.91)	117 (81.25)	χ^2^=10.98	401 (88.52)
			(1, *n*=453)	
Single	25 (8.09)	27 (18.75)	p=0.001	52 (11.48)
				
Currently living with*				
Alone	30 (9.71)	25 (17.73)		55 (12.22)
Spouse/Partner	247 (79.94)	78 (55.32)	χ^2^=41.47	325 (72.22)
Parent(s)	3 (0.97)	6 (4.26)	(6, *n*=450)	9 (2.00)
Friends	7 (2.27)	2 (1.42)	p<0.001	9 (2.00)
Other family members	13 (4.21)	9 (6.38)		22 (4.89)
Acquaintance	0 (0.00)	2 (1.42)		2 (0.44)
Other	9 (2.91)	19 (13.48)		28 (6.22)
				
**Any previous children**				
Yes	159 (51.46)	72 (50.00)	χ^2^=0.08	231 (50.99)
			(1, *n*=453)	
No	150 (48.54)	72 (50.00)	p=0.773	222 (49.01)
				
Previous miscarriage*				
No	230 (74.43)	86 (59.72)	χ^2^=10.08	316 (69.76)
			(1, *n*=453)	
Yes	79 (25.57)	58 (40.28)	p=0.002	137 (30.24)

**Gender of infant**				
Female	154 (50.00)	71 (49.31)	χ^2^=0.02	225 (49.78)
			(1, *n*=452)	
Male	154 (50.00)	73 (50.69)	p=0.891	227 (50.22)

**Infant GA at birth (in weeks)**				
Born at term (≥37)	288 (93.51)	137 (95.14)	χ^2^=0.47	425 (94.03)
			(1, *n*=452)	
Prematurely born (<37)	20 (6.49)	7 (4.86)	p=0.495	27 (5.97)
				
*Mean GA at birth ±SD (95% CI)*	*39.21 ±2.03 (38.98–39.43)*	*39.10 ±2.10 (38.76–39.45)*	***WMW****p=0.276*	*39.18 ±2.05*
			***T-test****p=0.606*	*(38.99 – 39.37)*

Pregnancy planned or unplanned*				
Planned	224 (72.49)	82 (56.94)	χ^2^=10.83	306 (67.55)
			(1, *n*=453)	
Unplanned	85 (27.51)	62 (43.06)	p=0.001	147 (32.45)

EPDS*				
Low symptoms (<13)	249 (83.56)	85 (59.44)	χ^2^=30.58	334 (75.74)
			(1, *n*=441)	
High symptoms (≥13 and including)	49 (16.44)	58 (40.56)	p<0.001	107 (24.26)
				
*Mean EPDS score ±SD (95% CI)*	*7.35 ±5.54 (6.72 – 7.98)*	*10.85 ±6.61 (9.75–11.94)*	***WMW****p<0.001*	*8.49 ±6.12*
			***T-test****p<0.001*	*(7.91 – 9.06)*

PDS®*				
*Mean PDS****®****score ±SD (95% CI)*	*1.72 ±5.14 (1.14–2.32)*	*8.20 ±11.41 (6.25–10.14)*	***WMW****p<0.001*	*3.77 ±8.24*
			***T-tes******t** p<0.001*	*(2.98 – 4.55)*

Pearson's chi-squared test was used to assess for population differences for categorical characteristics and Wilcoxon-Mann-Whitney test and t-test for continuous characteristics. The statistical tests used for continuous data have been highlighted in bold, with data presented in italics.

Note:  EPDS, Edinburgh postpartum bonding questionnaire. GA, gestational age. PBQ, postpartum bonding questionnaire. PDS®, Posttraumatic Stress Disorder Scale. SD, standard deviation. df, degrees of freedom. WMW, Wilcoxon-Mann-Whitney test. 95% CI, 95% confidence interval.

* p-value <0.05 thus significant difference between lifetime trauma experience and unexposed control.

a. In a relationship = In a relationship, cohabiting or married.

**Table 2:** Trauma categories reported via the composite trauma measurement.

**Trauma types**	**Reported trauma type alone (*n*)**	**Reported trauma type in addition to other categories (*n*)**	**Total reported (*n*)**
**Sexual abuse**	25	57	82
**Physical abuse**	14	55	69
**I**PV	18	25	43
**Child abuse**	1	35	36

Trauma categories reported by participants via the CAS(S), PDS**®** and SCID PTSD module and recorded in the composite trauma measurement.

Note: IPV, intimate partner violence.

Sexual abuse (both attempted and completed) by known or unknown perpetrator.

Physical abuse by known person or stranger (the latter only when categorised as traumatic).

Sexual, physical and non-physical abuse by an intimate partner.

Sexual, physical and non-physical abuse experienced in childhood (below the age of 16).

See Table 1 for sample characteristics. Women with experiences of lifetime trauma were younger (p=0.003), lower educated (p=0.016), more likely to be unemployed/unable to work (p<0.001), have a lower household income (p<0.001), be single (p=0.001), not to live with a partner (p<0.001), have had a previous miscarriage (p=0.002) and have a current unplanned pregnancy (p=0.001) than women without lifetime trauma experiences. In addition, higher antenatal EPDS scores were seen amongst those with lifetime trauma experience (p<0.001); mean (±SD) EPDS score was 10.85 (±6.61), compared to 7.35 (±5.54) of the non-exposed. Moreover, those with lifetime trauma experience were more likely to meet the clinical EPDS cut-off scores of ≥13 for probable depression (p<0.001). Higher PDS**®** scores were similarly noted for those with lifetime trauma experience (p<0.001); mean PDS**®** score of 8.20 (±11.41) compared to 1.72 (±5.14) of those without lifetime trauma experience. There was no difference in total PBQ score between those who had and had not experienced lifetime trauma (p=0.619); mean (±SD) PBQ score for those with experiences of lifetime trauma 7.78 (±7.99) compared to 7.17 (±6.76) among those without experiences of lifetime trauma.

### 3.3 Association between lifetime trauma exposure and PBQ scores

#### 3.3.1 Unadjusted linear regression – total PBQ scores

Table 3 presents the univariable regression models between exposures and outcome PBQ scores. Higher antenatal EPDS scores (depressive symptoms) on the continuous scale (0.24, 95% CI 0.13–0.36, p<0.001) were significantly associated with higher PBQ scores (increased perceptions of bonding difficulties). A scatter plot was used to visually examine the unadjusted relationship between antenatal EPDS scores and PBQ scores (see online supplementary Fig. 1). Being of Asian/Asian British descent (3.99, 95% CI 0.51–7.47, p=0.025) and having achieved a university/postgraduate degree (3.36, 95% CI 0.97–5.74, p=0.006) were significantly associated with higher PBQ scores (increased maternal perception of bonding difficulties). Being of black African, Caribbean or black British ethnicity (-3.21, 95% CI -4.76– -1.65, p<0.001) and having previous children (-1.76, 95% CI -3.15– -0.36, p=0.014) were associated with lower PBQ scores (decreased self-reported bonding difficulties). All other exposures, including lifetime trauma were not significantly associated with PBQ scores.

**Table 3:** Unadjusted and adjusted linear regression for the associations between lifetime trauma (exposure) and PBQ (outcome).

**Exposure**	**Unadjusted regression model**			**Adjusted regression model**		
	B-coefficient	95% CI	p-value	B-coefficient	95% CI	p-value
**Lifetime trauma**	-0.62	-2.13 – 0.90	0.423	-0.77	-2.36 – 0.82	0.343
Depressive symptoms (EPDS)*^,^**	0.24	0.13 – 0.36	<0.001*	0.33	0.17 – 0.50	<0.001**
**Posttraumatic symptoms severity (PDS®)**	0.02	-0.07 – 0.11	0.592	0.02	-0.12 – 0.17	0.772
**Maternal Age**	0.05	-0.08 – 0.18	0.422	0.67	-0.89 – 2.22	0.400
**Ethnicity**						
White	Reference			Reference		
Black African/Caribbean or black British*	-3.21	-4.76 – -1.65	<0.001*	-1.49	-3.11– 0.13	0.071
Asian/Asian British*	3.99	0.51 – 7.47	0.025*	-1.20	-3.64 – 1.25	0.337
Mixed/Multiple ethnic groups	-0.37	-3.94 – 3.20	0.840	0.72	-2.20 – 3.65	0.629
Arab/Other ethnicity	-2.11	-5.02 – 0.81	0.156	1.00	-2.23 – 4.06	0.567
**Education**						
None/School qualifications	Reference			Reference		
College/Diploma/ Higher certificate/ Training	1.68	-0.79 – 4.16	0.182	0.03	-3.01 – 3.07	0.987
University degree/ Postgraduate*	3.36	0.97 – 5.74	0.006*	1.27	-1.82 – 4.35	0.421
**Unemployed/Unable to work**	0.03	-1.83 – 1.89	0.977	-0.99	-3.15 – 1.17	0.368
**In a relationship**^**a**^	-0.62	-2.83 – 1.59	0.582	1.02	-2.25 – 4.29	0.541
Has previous children*	-1.76	-3.15 – -0.36	0.014*	-0.87	-2.32 – 0.57	0.263
**Male infant**	-0.17	-1.58 – 1.25	0.819	-0.38	-1.78 – 1.01	0.591
**Infant GA at birth**	-0.09	-0.43 – 0.26	0.622	-0.26	-0.59 – 0.08	0.131
**Unplanned pregnancy**	-0.96	-2.46 – 0.54	0.210	-0.28	-1.98 – 1.41	0.744

The results of the unadjusted and adjusted linear regressions to assess for association between lifetime trauma experience and postpartum bonding questionnaire (PBQ) score.

Note: EPDS, Edinburgh postpartum bonding questionnaire. GA, gestational age. PBQ, postpartum bonding questionnaire. PDS®, Posttraumatic Stress Disorder Scale. 95% CI, 95% confidence interval.

* p-value <0.05 thus significant for unadjusted regression.

** p-value <0.05 thus significant for adjusted regression.

a. In a relationship = In a relationship, cohabiting or married.

#### 3.3.2 Adjusted linear regression – lifetime trauma and total PBQ scores

Only increased antenatal EPDS scores remained significantly associated with PBQ scores after running the adjusted analysis. Increased EPDS antenatal scores (0.33, 95% CI 0.17–0.50, p<0.001) were associated with higher PBQ scores (increased risk of maternal perception of bonding difficulties). The sensitivity analysis revealed that reaching a clinical cut-off score on the antenatal EPDS of ≥13 (4.26, 95% CI 2.02–6.49, p<0.001) was also significantly associated with higher PBQ scores. Additionally, when further transforming the PBQ outcome to account for possible skewedness, the association between higher EPDS antenatal scores and higher PBQ scores remained (0.04, 95% CI 0.02–0.06, p<0.001). Antenatal EPDS reaching the clinical cut-off of ≥13 also remained significantly associated with higher PBQ scores (0.49, 95% CI 0.27–0.71, p<0.001).

#### 3.3.3 Unadjusted and adjusted linear regression – lifetime trauma and PBQ subscales

The results of the individual PBQ subscales, both unadjusted and adjusted can be found online within the supplementary tables S1 (impaired bonding), S2 (rejection and anger), S3 (anxiety about care) and S4 (risk of abuse).

Higher antenatal EPDS scores were associated with higher PBQ scores within the impaired bonding (0.18, 95% CI 0.08–0.28, p=0.001), rejection and anger (0.10, 95% CI 0.05–0.16, p<0.001) and anxiety about care (0.03, 95% CI 0.01-0.05, p=0.012) subscale.

Increased gestational age indicated lower PBQ impaired bonding scores (-0.22, 95% CI -0.43– -0.01, p=0.040). Being of black African/Caribbean or black British ethnicity was associated with lower PBQ scores in the rejection and anger subscale (-0.70, 95% CI -1.25– -0.16, p=0.012) and the anxiety about care subscale (-0.34, 95% CI, -0.53– -0.14, p=0.001). Being of Asian or Asian British ethnicity  was also associated with decreased scores in the anxiety about care subscale (-0.41, 95% CI -0.66– -0.15, p=0.002).

## 4 Discussion

The goal of this study was to assess the potential relationship between lifetime trauma experience and maternal perception of difficulties in bonding with her baby in a large cohort of expectant mothers recruited from an inner-city maternity service in South London, England. We were unable to find evidence to support our hypothesis of an association between a lifetime history of trauma experiences and maternal perceptions of bonding in our unadjusted or adjusted analyses. However, we did find that high levels of antenatal depressive symptoms were a predictor of maternal perceived mother-infant bonding difficulties.

The link between antenatal depressive symptoms and the mother's perception of difficulties in forming a relationship with her infant has been demonstrated in previous research studies (Borschmann et al., 2018; Field, 2010; Ohashi et al., 2016; Seng et al., 2013). Depression is known to impede on the sensitivity of a mother towards her infant and the establishment of sleep routines, positive interactions and enrichment activities (e.g., reading) postnatally, which may, in turn, impact the mother-infant relationship (Field, 2010). However, depression may also affect the development of a child in-utero. Through increased cortisol and serotonin reaching a developing fetus, depression may impact infant neurodevelopment (Davis and Sandman, 2010; Glover et al., 2018; Khalife et al., 2013). This has been theorised to be due to increased hormone production, alterations of its mediators and possibly alteration of gene expression through increased methylation (Capron et al., 2018; Conradt et al., 2013; Radtke et al., 2011). Maternal depressive symptomatology may further impact infant developmental outcomes via its association with preterm birth, low birthweight, maternal smoking and substance misuse in the perinatal period. All aforementioned are associated with significant infant morbidity and mortality (Alder et al., 2007; Glover et al., 2010).

In our study, higher EPDS scores antenatally were associated with mother-infant relational difficulties. Interestingly, reaching a score of 13 or higher, and thus reaching the threshold for probable clinical depression, had an increased β coefficient compared to the EPDS on a continuous scale, indicating greater association with perceived bonding difficulties. This implies not only that depressive symptoms at a clinical threshold in pregnancy may have a greater influence on self-reported bonding, but also that having higher scores on the EPDS, yet below clinical cut-off, are also indicative of potential perceived bonding difficulties. This repeats findings of greater psychosomatic symptoms (PTSD, major depressive disorder and anxiety) being increasingly predictive of negative maternal interaction with their child (Muzik et al., 2017; Seng et al., 2013). Yet lower levels of symptoms (not meeting clinical thresholds for possible disorder) are also associated with parenting stress, atypical parenting, infant regulation difficulties, decreased infant social engagement and perceived mother-infant bonding in both clinical and community populations (Burtchen et al., 2016; Tietz et al., 2014) and in populations with trauma experiences (Lehnig et al., 2019). When assessing the PBQ subscales separately, we found high antenatal depressive symptomatology to be associated with higher scores within the impaired bonding, rejection and anger and anxiety about care subscales; participants with increased antenatal depressive symptoms reported increasing feelings of emotional distance, rejection and anger towards their child and distress about care of their infant. Antenatal depressive symptomatology has been found to be associated with decreased PBQ scores in all subscales in a clinical and community sample (Garcia-Esteve et al., 2016). Our finding is also in direct contrast with some literature, indicating that those with pre-existing (antenatal) depressive symptoms in trauma exposed populations do not have associated impaired bonding (Lara-Cinisomo et al., 2018), but those who have co-morbid PTSD and depression may do (Seng et al., 2013). Increased infant gestational age (GA) at birth was associated with decreased scores of the impaired bonding subscale of the PBQ, indicating mothers reporting themselves to feel less emotionally distant towards their infant if born closer to 40 weeks GA. The environment in which a prematurely born neonate spends the first days to weeks of their lives could provide an explanation to this association. Prematurely born neonates often stay in neonatal intensive care units, a stressful and mechanical environment, impeding on skin-to-skin contact and perhaps a less nurturing environment for the mother-infant relationship than a home-setting (Ionio et al., 2016).

Any associations between overall perceived bonding difficulties and ethnicity were attenuated in the adjusted analysis accounting for depressive symptoms, PTSD symptom severity, trauma experience, sociodemographic and obstetric factors. However, being of black African/Caribbean or black British was protective for the “rejection and anger” and “anxiety about care” subscales. Consequently mothers with black African/Caribbean or black British ethnicities were less likely to report any anger or rejection feelings towards their child and possibly have increased confidence in care. Similarly women of Asian descent reported less issues regarding confidence in care. These results support initial findings from studies examining bonding behaviours across different ethnic groups in mothers receiving intensive psychiatric treatment (Loh and Vostanis, 2004; Sockol et al 2014), It has been posited that variations in bonding behaviours across ethnic groups may reflect differences in cultural norms related to adjustment in the perinatal period (Sockol et al 2014). Additional research would be beneficial to further explore these associations, including with observed mother-infant dyads to enrich the data.

We did not find an association between lifetime trauma experience and perceived maternal bonding difficulties. This is in contrast with previous findings which suggest childhood trauma experiences negatively affect self-reported mother-infant relationships (Choi et al., 2017; Savage et al., 2019), arguing possible parental relationship instability and altered parenting behaviour as underlying reasons. However, in line with our results, this association no longer remained when controlling for antenatal PTSD and/or depressive symptoms (Muzik et al., 2013; Muzik et al., 2017; Seng et al., 2013). Furthermore, symptoms of mental disorders may not be the only modifier of the association between trauma experience and perceived bonding difficulties. Maternal resilience and unresolved trauma (i.e. the degree to which past trauma exerts an ongoing influence on present experiences) may also play a role (Kim et al., 2014). The Adult Attachment Interview (George et al., 1984) is one example of how unresolved trauma can be assessed within a research context; unfortunately, the study on which this data analyses is based did not use any measures that directly examined unresolved trauma. Low maternal resilience and high depressive symptoms are associated with maternal childhood maltreatment and self-reported poor parenting (Martinez-Torteya et al., 2018a). Similarly, mothers whose functioning is still heavily affected by the experience of past trauma (unresolved trauma) have shown to be at increased risk of self-reported maternal-infant relationship difficulties. This is in comparison to mothers whose self-reported functioning is no longer affected by their trauma experience (resolved) or had no trauma exposure at all (Iyengar et al., 2014; Muzik et al., 2017). This may be due to sporadic lapses in daily discourse between mother and infant, which may occur for mothers with unresolved states of mind with respect to trauma (Lyons-Ruth et al., 2005; Madigan et al., 2006). These lapses could impact the infant-mother attachment as they may entail (unintended) inappropriate/intrusive behaviour from mother towards child (Iyengar et al., 2019). Moreover, infant cues of distress could potentially trigger memories of maternal trauma and impact maternal parenting behaviour amongst mothers with unresolved trauma (Hesse and Main, 2000; Iyengar et al., 2019; Madigan et al., 2006). Unresolved trauma would mean someone's functioning is still actively affected by their trauma, which could be reflected by increasing posttraumatic symptoms.

High posttraumatic symptom severity in mothers with lifetime interpersonal trauma history have shown an association with altered cortisol secretion and hypothalamic-pituitary-adrenal (HPA) dysregulation (Schechter et al., 2004; Ludmer et al., 2018). In a community sample of mothers, maternal maltreatment history was predictive of a disorganized infant-mother attachment for mothers with high cortisol secretion (Ludmer et al., 2018). Conversely, in a sample of mothers with lifetime interpersonal traumatic experiences and posttraumatic stress, low salivary cortisol was associated with increased observed atypical parenting behaviour/maternal disrupted communication with their infant (Schechter et al., 2004). Low salivary cortisol was used here as a marker of “maternal psychobiological dysregulation” and thought to be secondary to chronic activation of the HPA axis associated with longstanding posttraumatic stress. Atypical cortisol levels of mothers are associated with altered cortisol stress responses in their infants (Khoury et al., 2016). Higher maternal cortisol release has been associated with higher cortisol levels in infants around 17 months postnatally. Altered cortisol secretion in children is a risk factor for predisposing an infant to the development internalizing and externalizing psychopathology (Goodyer et al., 2000; Kamin and Kertes, 2017).

In our study increasing posttraumatic symptoms was reflected by higher PDS® scores, but an association between posttraumatic stress symptom severity and negative maternal perceptions of the mother-infant relationship was not found. Despite trauma being common, our study indicates that trauma experience does not appear to limit a mother in forming a bond with her child. The psychopathology following trauma experience and possible consequent mother-infant relationship difficulties is complex and modifiable (Morelen et al., 2018). For example, one can treat the arising mental disorder symptoms and this could prevent against consequent intrusive parenting behaviour (Stephenson et al., 2018). Similarly, parenting interventions could be provided to aid the mother-infant relationship (Guedeney et al., 2014; Stephenson et al., 2018). Likewise, genetics may play a role in both maternal and infant vulnerability to relationship difficulties. One such influential gene site being OXTR, where variations in genotype may influence parenting behaviour and possibly moderate the impact of maternal trauma on the mother-infant relationship (Ludmer et al., 2018).

The aforementioned points expose how much there is left to elucidate regarding the complex relationship between maltreatment history and perceived mother-infant relationships. Perhaps it would be of great interest to explore its underlying causative pathways further in future research, especially in a cohort considering the independent impact of child- and adulthood trauma experience.

### 4.1 Strengths and Limitations

The study consisted of an ethnically and socio-economically diverse population. This allows for the findings to be representative of the base populations and other populations of similar demographics and is suggestive of the external validity. Moreover, the study used validated measures of trauma experience, antenatal depression, antenatal posttraumatic symptoms and maternal perception of bonding with her infant, with a large number of participants answering the SCID interview questions regarding trauma and symptoms of mental disorders. Furthermore, the other study variables used were comprehensive, used validated structured measures where possible and based on previous literature. The weighting for both stratified sampling and missingness and the transformation of the PBQ scores to adjust for possible skewing of data, allowed for a more accurate assessment of the influence of trauma on self-reported bonding and any other potential adverse exposures, such as antenatal depressive symptoms. Finally, the larger population size of the cohort enhanced the power of the findings of the study.

However, albeit a diverse population, the study participants were recruited from only one hospital in South London. Additionally, the composite lifetime trauma measure may have under-reported the extent of lifetime trauma experiences as no specific validated measure of childhood abuse was administered, and birth-related trauma was also not specifically measured. Similarly, we did not make a comparison of interpersonal versus non-interpersonal traumas, yet there is evidence that interpersonal trauma is more impactful on mental health (Kessler et al., 2017). In addition, due to small sample sizes with respect to distinct timings (i.e. child or adulthood) and type of trauma (i.e. sexual or physical abuse) we were unable to examine the influence of individual trauma categories (i.e., intimate partner violence or childhood abuse). Our data highlighted that many participants had experiences of traumas in multiple categories, both in child- and adulthood, which are often interrelated (Barnes et al., 2009; Dias et al., 2017; Frederick and Goddard, 2008) and mediate impact (Choi and Sikkema, 2015). Exploring individual trauma types may be of interest in future study to elucidate if recency and type impacts maternal health and mother-infant relations differently.

Other limiting factors include the lack of measures of child temperament and paternal factors (Edhborg et al., 2005; Kerstis et al., 2016). The PBQ is a self-reported measure and only addresses maternal perception of the relationship with her child. We did not include an observational outcome of the mother-infant relationship in our analysis. This is a weakness as there is some evidence to suggest that maternal perceptions may not align with video observations of the mother-infant relationship (Nath et al., 2019). Additionally, we were unable to measure maternal insightfulness (Martinez-Torteya et al., 2018b) and resilience (Martinez-Torteya et al., 2018a; Muzik et al., 2017; Sexton et al., 2017), the influence of which may be interesting to explore further in future study.

### 4.2 Implications

This study highlights the impact antenatal depressive symptoms may have on a mother's perception of her relationship with her baby at three months after birth. Depressive symptoms within the perinatal period are currently screened for in the UK by midwives at the first antenatal appointment. Based on the findings of this study, identification of increased depressive symptoms in the early antenatal period may also provide an indicator of subsequent difficulties for the mother in bonding with her baby in the early postnatal period. Identifying antenatal depressive symptoms could allow for early treatment, which may also prevent perceived difficulties in mother-infant bonding and subsequent negative effects on infant outcomes in the presence of bonding difficulties (Fisher et al., 2011; Guedeney et al., 2014; Stephenson et al., 2018). A reassuring finding from this work is that despite trauma experience being common amongst our cohort, it did not appear to limit the early bonds a mother forms with her child.

## 5 Conclusion

The experience of trauma is common, yet its effects are diverse and can range from no consequences to a devastating impact on mental health and parental functioning. In our study there was an association between a mother's lifetime experience of trauma and depressive symptoms in pregnancy. However, there was no direct effect of lifetime trauma experience on the mother's perception of difficulties in bonding with her baby. Increased antenatal depressive symptoms were found to be associated with increased perceived mother-infant bonding difficulties. This association remained even when controlling for the experience of lifetime trauma, antenatal posttraumatic symptoms and various sociodemographic influences. Consequently, when a pregnant woman is identified to have depressive symptoms, she may benefit from support in bonding with her infant both ante- and postnatally.

## Ethics approval

This was a secondary data analysis of already collected data by Howard et al. (2018) for which ethics approval was already gained by the National Research Ethics Service, London Committee – Camberwell St Giles (ref no 14/LO/0075).

## Funding

This research was funded by the National Institute for Health Research (NIHR) under the Programme Grants for Applied Research programme (ESMI Programme: grant reference number RP-PG-1210-12002) and the National Institute for Health Research (NIHR) / Wellcome Trust Kings Clinical Research Facility and the NIHR Biomedical Research Centre and Dementia Unit at South London and Maudsley NHS Foundation Trust and Kings College London. The study team acknowledges the study delivery support given by the South London Clinical Research Network. Professor L. M. Howard also had salary support from an NIHR Research Professorship (NIHR-RP-R3-12-011). The views expressed are those of the author(s) and not necessarily those of the NHS, the NIHR, or the Department of Health and Social Care.

## Contributors

T. A. Kolk carried out statistical analysis, data interpretation, drafted and edited the manuscript. Dr. S. Nath supported the statistical analysis and reviewed and edited the manuscript. Dr. K. Trevillion aided in the literature search and drafting of the manuscript introduction and editing of the manuscript. Dr. S. Pawlby critically reviewed the analysis and manuscript. Dr. G. Lockwood-Estrin reviewed the manuscript and created the composite abuse measurement used for this study. Professor L. M. Howard designed the original WENDY study, of which this study is a secondary analysis, and was involved with the reviewing and editing of the manuscript. All authors have seen and approved the manuscriptsubmitted.

## Declaration of Competing Interest

None.

## Appendix Supplementary materials

Image, application 1
