# Supplementary material for: The association between maternal lifetime interpersonal trauma experience and perceived mother-infant bonding
Source: J Affect Disord. 2021 Nov 1;294:117–27. doi: 10.1016/j.jad.2021.06.069 (PMC8424749; doi:10.1016/j.jad.2021.06.069)
Supplement: Supplementary file 1 [file mmc1.docx]

| **Table S.1** | | **Unadjusted and adjusted linear regression for the associations between lifetime trauma (exposure) and PBQ impaired bonding subscale (outcome)** | | | | | | | | | | | | |  |
| --- | --- | --- | --- | --- | --- | --- | --- | --- | --- | --- | --- | --- | --- | --- | --- |
| **Exposure** | | | **Unadjusted regression model** | | | |  | | **Adjusted regression model** | | | | | |  |
|  |  |  | B-coefficient | 95% CI | | p-value |  | | B-coefficient | | 95% CI | | p-value | |  |
| Lifetime trauma | | | -0.30 | | -1.18 – 0.59 | 0.513 | |  | | -0.51 | | -1.49 – 0.46 | | 0.302 | |
| Depressive symptoms (EPDS)*^,^ ** | | | 0.12 | | 0.06 – 0.19 | <0.001* | |  | | 0.18 | | 0.08 – 0.28 | | 0.001** | |
| Posttraumatic symptoms severity (PDS**^®^**) | | | 0.02 | | -0.03 – 0.07 | 0.441 | |  | | 0.01 | | -0.08 – 0.09 | | 0.852 | |
| Maternal Age | | | 0.01 | | -0.07 – 0.09 | 0.822 | |  | | 0.55 | | -0.46 – 1.55 | | 0.286 | |
| Ethnicity |  | |  |  | |  | |  | |  | |  | |  | |
| White | | | Reference | | | | |  | | Reference | | | | | |
| Black African/Caribbean or black British* | | | -1.22 | | -2.14 – -0.30 | 0.009* | |  | | -0.36 | | -1.48 – 0.77 | | 0.532 | |
| Asian/Asian British* | | | 2.97 | | 0.90 – 5.03 | 0.005* | |  | | 0.14 | | -1.38 – 1.65 | | 0.859 | |
| Mixed/Multiple ethnic groups | | | -0.17 | | -2.29 – 1.95 | 0.874 | |  | | 0.41 | | -1.63 – 2.45 | | 0.691 | |
| Arab/Other ethnicity | | | -1.06 | | -2.79 – 0.67 | 0.229 | |  | | 0.96 | | -1.34 – 3.27 | | 0.411 | |
| Education |  | |  |  | |  | |  | |  | |  | |  | |
| None/School qualifications | | | Reference | | | | |  | | Reference | | | | | |
| College/Diploma/ Higher certificate/ Training | | | 0.72 | | -0.75 – 2.19 | 0.338 | |  | | 0.28 | | -1.79 – 2.34 | | 0.792 | |
| University degree/ Postgraduate | | | 1.22 | | -0.19 – 2.63 | 0.090 | |  | | 0.52 | | -1.57 – 2.62 | | 0.624 | |
| Unemployed/Unable to work | | | 0.64 | | -0.45 – 1.73 | 0.250 | |  | | -0.43 | | -1.70 – 0.85 | | 0.512 | |
| In a relationship^a^ | | | 0.40 | | -0.90 – 1.70 | 0.546 | |  | | 1.11 | | -0.88 – 3.09 | | 0.275 | |
| Has previous children | | | -0.78 | | -1.61 – 0.04 | 0.062 | |  | | -0.51 | | -1.37 – 0.35 | | 0.245 | |
| Male infant | | | -0.04 | | -0.87 – 0.79 | 0.931 | |  | | -0.05 | | -0.90 – 0.80 | | 0.904 | |
| Infant GA at birth** | | | -0.10 | | -0.30 – 0.10 | 0.324 | |  | | -0.22 | | -0.43 – -0.01 | | 0.040** | |
| Unplanned pregnancy | | | -0.06 | | -0.94 – 0.83 | 0.900 | |  | | -0.08 | | -1.20 – 1.05 | | 0.894 | |

**Online Supplementary table 1**:
The results of the unadjusted and adjusted linear regressions to assess for association between lifetime trauma experience and postpartum bonding questionnaire (PBQ) impaired bonding subscale.
Note: EPDS, Edinburgh postpartum bonding questionnaire. GA, gestational age. PBQ, postpartum bonding questionnaire. PDS**^®^**, Posttraumatic Stress Disorder Scale. 95% CI, 95% confidence interval.
* p-value <0.05 thus significant for unadjusted regression.
** p-value <0.05 thus significant for adjusted regression.
a. In a relationship = In a relationship, cohabiting or married.

| **Table S.2** | | **Unadjusted and adjusted linear regression for the associations between lifetime trauma (exposure) and PBQ rejection and anger subscale (outcome)** | | | | | | | | | | | | |  |
| --- | --- | --- | --- | --- | --- | --- | --- | --- | --- | --- | --- | --- | --- | --- | --- |
| **Exposure** | | | **Unadjusted regression model** | | | |  | | **Adjusted regression model** | | | | | |  |
|  |  |  | B-coefficient | 95% CI | | p-value |  | | B-coefficient | | 95% CI | | p-value | |  |
| Lifetime trauma | | | -0.16 | | -0.66 – 0.35 | 0.542 | |  | | -0.22 | | -0.86 – 0.42 | | 0.495 | |
| Depressive symptoms (EPDS)*^,^ ** | | | 0.08 | | 0.04 – 0.12 | <0.001* | |  | | 0.10 | | 0.05 – 0.16 | | <0.001** | |
| Posttraumatic symptoms severity (PDS**^®^**) | | | 0.01 | | -0.02 – 0.04 | 0.646 | |  | | 0.01 | | -0.05 – 0.07 | | 0.688 | |
| Maternal Age | | | 0.04 | | -0.00 – 0.08 | 0.071 | |  | | 0.06 | | -0.46 – 0.57 | | 0.824 | |
| Ethnicity |  | |  |  | |  | |  | |  | |  | |  | |
| White | | | Reference | | | | |  | | Reference | | | | | |
| Black African/Caribbean or black British*, ** | | | -1.18 | | -1.70 – -0.66 | <0.001* | |  | | -0.70 | | -1.25 – -0.16 | | 0.012** | |
| Asian/Asian British | | | 0.66 | | -0.51 – 1.83 | 0.267 | |  | | -0.53 | | -1.36 – 0.30 | | 0.211 | |
| Mixed/Multiple ethnic groups | | | -0.34 | | -1.54 – 0.86 | 0.574 | |  | | 0.34 | | -0.63 – 1.32 | | 0.487 | |
| Arab/Other ethnicity | | | -0.97 | | -1.94 – 0.01 | 0.053 | |  | | -0.33 | | -1.23 – 0.56 | | 0.465 | |
| Education |  | |  |  | |  | |  | |  | |  | |  | |
| None/School qualifications | | | Reference | | | | |  | | Reference | | | | | |
| College/Diploma/ Higher certificate/ Training | | | 0.63 | | -0.20 – 1.46 | 0.135 | |  | | -0.32 | | -1.31 – 0.68 | | 0.533 | |
| University degree/ Postgraduate* | | | 1.34 | | 0.55 – 2.14 | 0.001* | |  | | 0.31 | | -0.71 – 1.33 | | 0.550 | |
| Unemployed/Unable to work | | | -0.50 | | -1.12 – 0.12 | 0.117 | |  | | -0.30 | | -0.93 – 0.33 | | 0.343 | |
| In a relationship ^a^ | | | -0.48 | | -1.22 – 0.26 | 0.204 | |  | | 0.01 | | -1.00 – 1.02 | | 0.989 | |
| Has previous children | | | -0.38 | | -0.85 – 0.10 | 0.118 | |  | | -0.15 | | -0.74 – 0.43 | | 0.602 | |
| Male infant | | | -0.24 | | -0.72 – 0.23 | 0.314 | |  | | -0.38 | | -0.92 – 0.16 | | 0.167 | |
| Infant GA at birth | | | -0.03 | | -0.08 – 0.15 | 0.560 | |  | | 0.03 | | -0.09 – 0.15 | | 0.639 | |
| Unplanned pregnancy | | | -0.49 | | -0.99 – 0.02 | 0.058 | |  | | -0.34 | | -0.95 – 0.27 | | 0.276 | |

**Online Supplementary table 2**: The results of the unadjusted and adjusted linear regressions to assess for association between lifetime trauma experience and postpartum bonding questionnaire (PBQ) rejection and anger subscale.
Note: EPDS, Edinburgh postpartum bonding questionnaire. GA, gestational age. PBQ, postpartum bonding questionnaire. PDS**^®^**, Posttraumatic Stress Disorder Scale. 95% CI, 95% confidence interval.
* p-value <0.05 thus significant for unadjusted regression.
** p-value <0.05 thus significant for adjusted regression.
a. In a relationship = In a relationship, cohabiting or married.

| **Table S.3** | | **Unadjusted and adjusted linear regression for the associations between lifetime trauma (exposure) and PBQ anxiety about care subscale (outcome)** | | | | | | | | | | | | |  |
| --- | --- | --- | --- | --- | --- | --- | --- | --- | --- | --- | --- | --- | --- | --- | --- |
| **Exposure** | | | **Unadjusted regression model** | | | |  | | **Adjusted regression model** | | | | | |  |
|  |  |  | B-coefficient | 95% CI | | p-value |  | | B-coefficient | | 95% CI | | p-value | |  |
| Lifetime trauma | | | -0.10 | | -0.33 – 0.13 | 0.385 | |  | | -0.07 | | -0.30 – 0.15 | | 0.530 | |
| Depressive symptoms (EPDS)*^,^ ** | | | 0.03 | | 0.01 – 0.04 | 0.005* | |  | | 0.03 | | 0.01 – 0.05 | | 0.012** | |
| Posttraumatic symptoms severity (PDS**^®^**) | | | < -0.01 | | -0.01 – 0.01 | 0.910 | |  | | 0.01 | | -0.01 – 0.03 | | 0.402 | |
| Maternal Age | | | < 0.01 | | -0.02 – 0.02 | 0.792 | |  | | 0.03 | | -0.14 – 0.21 | | 0.719 | |
| Ethnicity |  | |  |  | |  | |  | |  | |  | |  | |
| White | | | Reference | | | | |  | | Reference | | | | | |
| Black African/Caribbean or black British*, ** | | | -0.35 | | -0.59 – -0.11 | 0.004* | |  | | -0.34 | | -0.53 – -0.14 | | 0.001** | |
| Asian/Asian British ** | | | 0.38 | | -0.16 – 0.92 | 0.164 | |  | | -0.41 | | -0.66 – -0.15 | | 0.002** | |
| Mixed/Multiple ethnic groups | | | < -0.01 | | -0.56 – 0.55 | 0.989 | |  | | 0.07 | | -0.61 – 0.48 | | 0.815 | |
| Arab/Other ethnicity | | | -0.06 | | -0.52 – 0.39 | 0.782 | |  | | -0.09 | | -0.47 – 0.30 | | 0.666 | |
| Education |  | |  |  | |  | |  | |  | |  | |  | |
| None/School qualifications | | | Reference | | | | |  | | Reference | | | | | |
| College/Diploma/ Higher certificate/ Training | | | 0.15 | | -0.23 – 0.53 | 0.427 | |  | | -0.01 | | -0.34 – 0.32 | | 0.951 | |
| University degree/ Postgraduate* | | | 0.44 | | 0.08 – 0.81 | 0.018* | |  | | 0.22 | | -0.10 – 0.55 | | 0.181 | |
| Unemployed/Unable to work | | | -0.03 | | -0.32 – 0.25 | 0.817 | |  | | < -0.01 | | -0.02 – 0.99 | | 0.951 | |
| In a relationship ^a^ | | | -0.27 | | -0.60 – 0.07 | 0.116 | |  | | 0.09 | | -0.28 – 0.46 | | 0.647 | |
| Has previous children* | | | -0.32 | | -0.53 – -0.11 | 0.003* | |  | | -0.03 | | -0.26 – 0.21 | | 0.832 | |
| Male infant | | | -0.09 | | -0.12 – 0.31 | 0.397 | |  | | 0.01 | | -0.21 – 0.22 | | 0.947 | |
| Infant GA at birth | | | < -0.01 | | -0.06 – 0.05 | 0.910 | |  | | -0.01 | | -0.06 – 0.04 | | 0.707 | |
| Unplanned pregnancy* | | | -0.25 | | -0.48 – -0.02 | 0.030* | |  | | 0.03 | | -0.19 – 0.25 | | 0.797 | |

**Online Supplementary table 3**:
The results of the unadjusted and adjusted linear regressions to assess for association between lifetime trauma experience and postpartum bonding questionnaire (PBQ) anxiety about care subscale.
Note: EPDS, Edinburgh postpartum bonding questionnaire. GA, gestational age. PBQ, postpartum bonding questionnaire. PDS**^®^**, Posttraumatic Stress Disorder Scale. 95% CI, 95% confidence interval.
* p-value <0.05 thus significant for unadjusted regression.
** p-value <0.05 thus significant for adjusted regression.
a. In a relationship = In a relationship, cohabiting or married.

| **Table S.4** | | **Unadjusted and adjusted linear regression for the associations between lifetime trauma (exposure) and PBQ risk of abuse subscale (outcome)** | | | | | | | | | | | | |  |
| --- | --- | --- | --- | --- | --- | --- | --- | --- | --- | --- | --- | --- | --- | --- | --- |
| **Exposure** | | | **Unadjusted regression model** | | | |  | | **Adjusted regression model** | | | | | |  |
|  |  |  | B-coefficient | 95% CI | | p-value |  | | B-coefficient | | 95% CI | | p-value | |  |
| Lifetime trauma | | | -0.04 | | -0.25 – 0.17 | 0.712 | |  | | 0.04 | | -0.22 – 0.29 | | 0.782 | |
| Depressive symptoms (EPDS) | | | 0.01 | | -0.01 – 0.03 | 0.220 | |  | | 0.02 | | -0.01 – 0.05 | | 0.102 | |
| Posttraumatic symptoms severity (PDS**^®^**) | | | < -0.01 | | -0.01 – 0.01 | 0.893 | |  | | -0.01 | | -0.03 – 0.01 | | 0.430 | |
| Maternal Age | | | < 0.01 | | -0.02 – 0.02 | 0.805 | |  | | 0.03 | | -0.20 – 0.26 | | 0.812 | |
| Ethnicity |  | |  |  | |  | |  | |  | |  | |  | |
| White | | | Reference | | | | |  | | Reference | | | | | |
| Black African/Caribbean or black British* | | | -0.47 | | -0.69 – -0.25 | < 0.001* | |  | | -0.10 | | -0.53 – 0.34 | | 0.667 | |
| Asian/Asian British | | | -0.03 | | -0.52 – 0.46 | 0.899 | |  | | -0.41 | | -0.81 – -0.01 | | 0.057 | |
| Mixed/Multiple ethnic groups | | | 0.14 | | -0.37 – 0.64 | 0.600 | |  | | 0.03 | | -0.48 – 0.53 | | 0.916 | |
| Arab/Other ethnicity | | | -0.03 | | -0.44 – 0.38 | 0.880 | |  | | 0.37 | | -0.08 – 0.82 | | 0.106 | |
| Education |  | |  |  | |  | |  | |  | |  | |  | |
| None/School qualifications | | | Reference | | | | |  | | Reference | | | | | |
| College/Diploma/ Higher certificate/ Training | | | 0.19 | | -0.17 – 0.54 | 0.299 | |  | | 0.07 | | -0.60 – 0.75 | | 0.827 | |
| University degree/ Postgraduate* | | | 0.36 | | 0.02 – 0.70 | 0.038* | |  | | 0.21 | | -0.47 – 0.88 | | 0.547 | |
| Unemployed/Unable to work | | | -0.09 | | -0.35 – 0.17 | 0.493 | |  | | -0.26 | | -0.60 – 0.08 | | 0.135 | |
| In a relationship ^a^ | | | -0.30 | | -0.60 – 0.01 | 0.053 | |  | | -0.19 | | -0.70 – 0.32 | | 0.460 | |
| Has previous children* | | | -0.30 | | -0.49 – -0.10 | 0.003* | |  | | -0.19 | | -0.44 – 0.06 | | 0.141 | |
| Male infant | | | 0.01 | | -0.19 – 0.21 | 0.930 | |  | | 0.05 | | -0.19 – 0.28 | | 0.701 | |
| Infant GA at birth | | | -0.02 | | -0.06 – 0.03 | 0.491 | |  | | -0.06 | | -0.12 – 0.01 | | 0.095 | |
| Unplanned pregnancy | | | -0.19 | | -0.40 – -0.02 | 0.081 | |  | | 0.10 | | -0.22 – 0.43 | | 0.532 | |

**Online Supplementary table 4**:
The results of the unadjusted and adjusted linear regressions to assess for association between lifetime trauma experience and postpartum bonding questionnaire (PBQ) risk of abuse subscale.

Note: EPDS, Edinburgh postpartum bonding questionnaire. GA, gestational age. PBQ, postpartum bonding questionnaire. PDS**^®^**, Posttraumatic Stress Disorder Scale. 95% CI, 95% confidence interval.
* p-value <0.05 thus significant for unadjusted regression.
** p-value <0.05 thus significant for adjusted regression.
a. In a relationship = In a relationship, cohabiting or married.

**Online Supplementary figure 1**: A scatterplot to show the association between increased PBQ total scores and antenatal depressive symptoms (EPDS score).


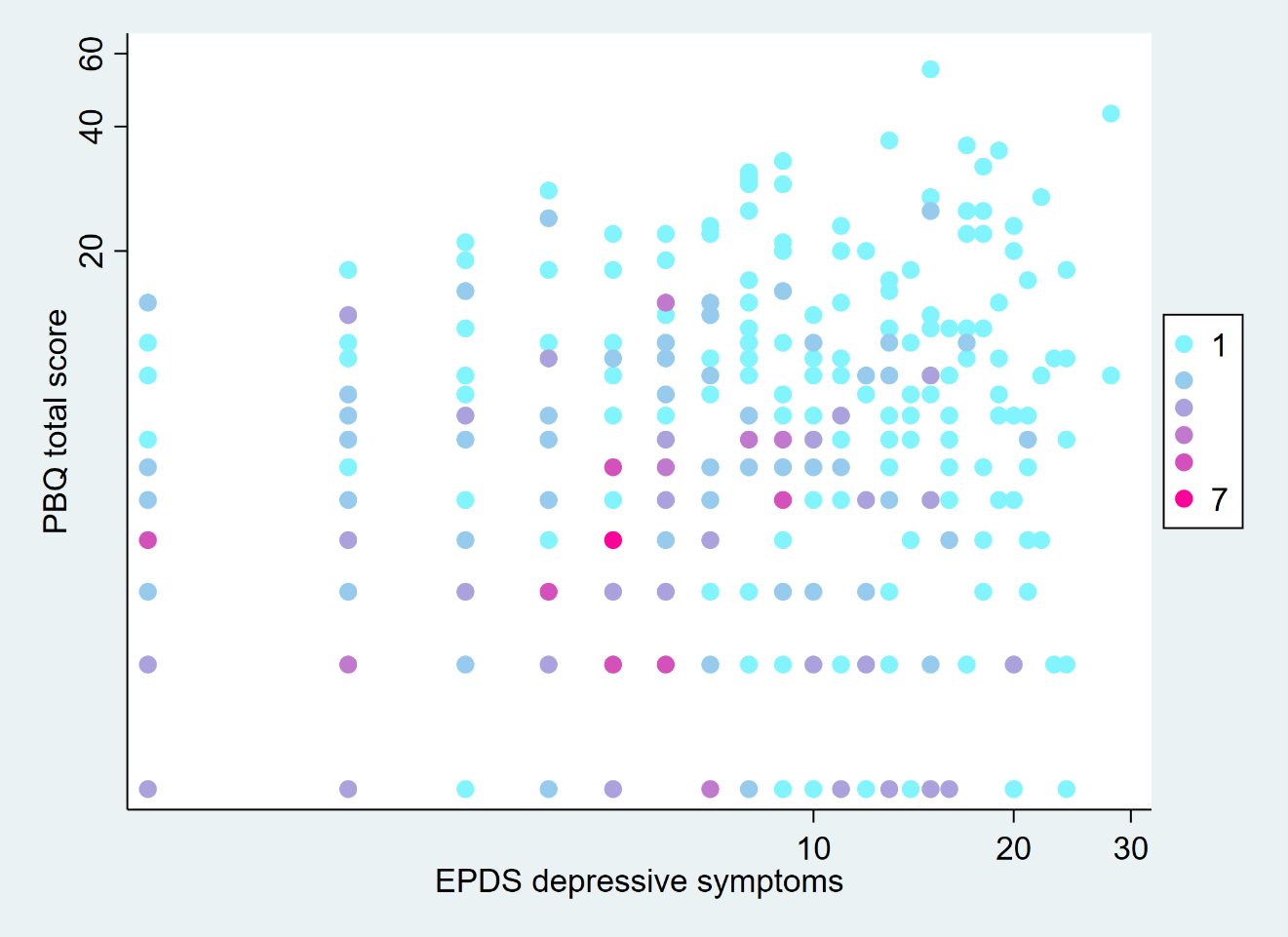


Note: The colour scale in the Legend is used to illustrate the count of the observations that share the same values on the EPDS depressive symptoms and PBQ total score scales. The gradient of colors in the Legend, between 1 and 7, visually display the density of the distribution. The plot image helps to visualize the findings of the statistical test, which show that higher antenatal depressive symptoms on the EPDS scale are associated with greater bonding difficulties as measured by the PBQ.
